# The effect of age on the attachment ability of stick insects (Phasmatodea)

**DOI:** 10.3762/bjnano.15.72

**Published:** 2024-07-15

**Authors:** Marie Grote, Stanislav N Gorb, Thies H Büscher

**Affiliations:** 1 Functional Morphology and Biomechanics, Kiel University, Am Botanischen Garten 1-9, D-24118 Kiel, Germany

**Keywords:** adhesion, attachment pads, friction, locomotion, morphology, material properties, wear

## Abstract

Many insect species have found their way into ageing research as small and easy-to-keep model organisms. A major sign of ageing is the loss of locomotory functions due to neuronal disorders or tissue wear. Soft and pliable attachment pads on the tarsi of insects adapt to the substrate texture to maximize their real contact area and, thereby, generate attachment during locomotion. In the majority of stick insects, adhesive microstructures covering those pads support attachment. Stick insects do not molt again after reaching the imaginal stage; hence, the cuticle of their pads is subject to continuous ageing. This study aims to quantify how attachment ability changes with age in the stick insect *Sungaya aeta* Hennemann, 2023 and elucidate the age effects on the material and microstructure of the attachment apparatus. Attachment performance (adhesion and friction forces) on substrates with different roughnesses was compared between two different age groups, and the change of attachment performance was monitored extending over a larger time frame. Ageing effects on the morphology of the attachment pads and the autofluorescence of the cuticle were documented using light, scanning electron, and confocal laser scanning microscopy. The results show that both adhesion and friction forces decline with age. Deflation of the pads, scarring of the cuticle, and alteration of the autofluorescence, likely indicating stiffening of the cuticle, were observed to accumulate over time. This would reduce the attachment ability of the insect, as pads lose their pliant properties and cannot properly maintain sufficient contact area with the substrate.

## Introduction

Ageing inexorably affects most living organisms, does not exclude insects, and makes different organs or tissues susceptible to wear or fatigue of material [1]. Research on the time-dependent decline of body functions has often been focused on vertebrates, especially mammals, but insects have found their way into ageing research as well [2–4]. They are easy to maintain and have a short lifespan, and changes in their exoskeleton can be easily observed [5]. The process of ageing has been explored most thoroughly in *Drosophila melanogaster* (Meigen, 1830) and other dipterans, often with special regards to flight [2,4,6–8]. Other studies on ageing in insects included economically or ecologically important species, such the silkworm moth *Bombyx mori* (L., 1758) or ants [4]. One difficulty of measuring age-dependent functional decay is finding feasible methods to investigate underlying material fatigue.

One functional system affected by age and of concern for locomotion and, hence, for the survival of individual insects is the attachment system. Two different attachment mechanisms evolved in insects, namely, hairy pads consisting of flexible setae, which adapt to the surface topography, and smooth pads possessing a soft and deformable cuticle to comply with the substrate profile [9]. Both pad types, hairy and smooth, aim to maximize contact area with the substrate as the contact area of the pad is proportional to adhesion [10–12]. For rough substrates, the pads are complemented by a pair of rigid claws used for friction interlocking with surface asperities and ensuring attachment, but claws perform poorly on smooth surfaces [13]. The ability to attach to various surfaces is helpful for climbing animals [11,14], and adapting to the quality of the substrate is especially important for motile animals, which may come into contact with different surfaces, such as plants [9,11,15]. In ageing cockroaches, attachment pad discoloration and increased stiffness are accompanied by the decreased ability to climb an incline [5,16]. Zhou et al. [17] found stiffer and darker “scars” on the pads of aged cockroaches, most likely due to accumulated damage, resulting in the pads not being as compliant as in younger cockroaches. Slifer [18] made similar observations in locusts walking on abrasive sandpaper, leading to the formation of scars in older animals. Scars and stiffened cuticle likely conflict with the functionality of soft adhesive pads as the contact formation of the cuticle is hampered by the reduced material compliance.

Phasmatodea, also known as stick and leaf insects, are a lineage of large terrestrial insects encompassing around 3500 described species thriving in different habitats [19–20]. They are exclusively herbivorous and camouflage themselves as twigs, leaves, or bark [19,21]. As phasmids are slow and most of them wingless or unable to fly, they adapted strongly to their local environment [11,19,22–23]. Phasmids have evolved considerably depending on plants since pre-angiosperm times [24]. As plants display a huge range of different surface characteristics [25–28], the diversity of microstructures on phasmatodean attachment pads is assumed to result from adaptations towards these plant surfaces [23,29]. Phasmids possess smooth adhesive pads on their tarsomeres, the euplantulae, and one larger pad at the pretarsus, the arolium [30]. Investigations of the specific functionality of both euplantulae and the arolium by Labonte and Federle [31] have shown that the arolium and euplantulae each perform different tasks. The arolium is used while climbing upside down, whereas the euplantulae generate friction and are used in upright walking. Phasmid euplantulae are covered with different surface microstructures that are likely adapted to specific surface parameters in their environments [32–34]. It has been shown that nubby euplantulae perform better on rough surfaces whereas pads without protrusions perform better on smooth surfaces [35]. Experimental studies concerning the attachment ability of phasmids investigated various functions of this system and how it changes under certain conditions, such as substrate geometry [36], the presence or absence of claws [37], different surface characteristics of substrates [33,38–39], and the combined effect with pad fluids [40]. For these animals, whose lives strongly depend on plants for camouflage and nutrition, attachment to the plant surface is crucial for survival [11,14,21].

Their life history makes phasmids interesting study subjects for ageing research, as this lineage represents some of the largest insects known and species that have a prolonged life expectancy of up to three years after imaginal molt [41]. After this last molt, phasmids do not molt anymore and, hence, their cuticle is subject to continuous ageing. So far, representatives of Phasmatodea and their adhesive systems have not been investigated with regards to ageing. Nevertheless, the stiffness of the cuticle of these organs and the internal pressure are important for the functionality and likely susceptible to decay during ageing [42–43]. We investigated the change in attachment ability and tarsal morphology in the species *Sungaya aeta* Hennemann, 2023 (Heteropterygidae). Members of Heteropterygidae can reach impressive life expectancies [41,44], with anecdotal reports extending over five years. The change in attachment performance was quantified through attachment force measurements. Because of the different properties of arolium and euplantulae [31,33], the attachment forces of whole animals were compared in two directions. The pull-off force was measured perpendicular to the substrate, and the traction force parallel to the substrate, to assess the ability of the insect to attach itself in the respective direction and evaluate potential differences arising from performance decay of either of the two components of the overall attachment system.

The aim of this study was to answer the following research questions: (1) Does the attachment ability of older animals differ from that of younger animals? (2) Do pull-off and traction forces on the same substrates change during age? (3) Does the morphology of the tarsus and the attachment pads differ between younger and older animals?

## Materials and Methods

### 1 Animals and experimental conditions

**Figure 1:**
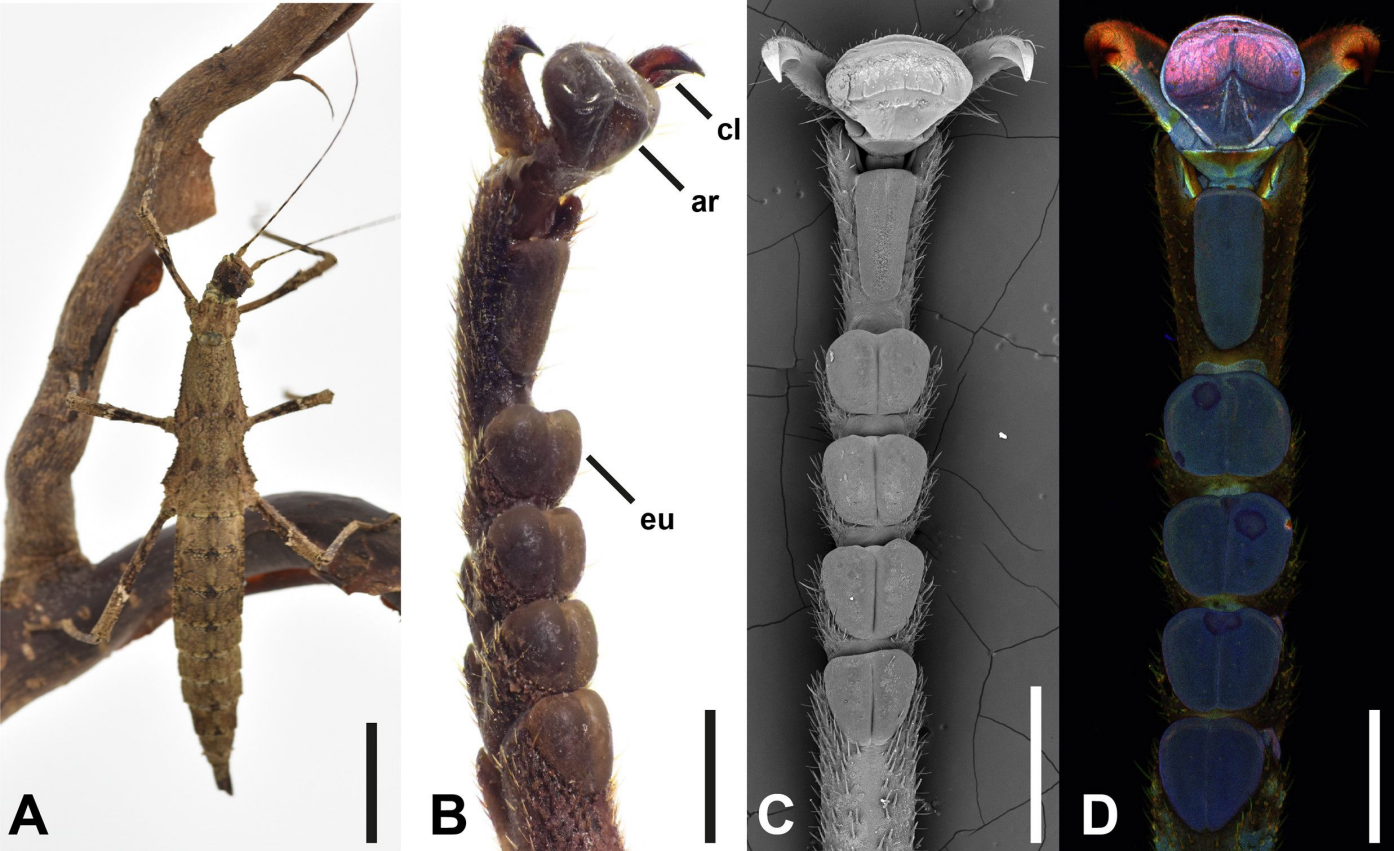
Focal species. (A) Adult female of *Sungaya aeta*. (B–D) Overviews of the tarsal morphology obtained with different techniques: (B) stereomicroscope, (C) scanning electron microscope, and (D) confocal laser scanning microscope. ar, arolium; cl, claw; eu, euplantula. Scale bars: (A) 2 cm, (B–D) 1 mm.

Two groups of 15 adult females per group of *Sungaya aeta* Hennemann, 2023 (Phasmatodea: Heteropterygidae, Figure 1) were selected from laboratory stock (Department for Functional Morphology and Biomechanics, Kiel University), kept under ambient conditions, and fed with fresh blackberry leaves ad libitum. This species was previously referred to as *Sungaya inexpectata* Zompro, 1996, until the original population of this widespread culture stock from Bataan Province, Ilanin Forest, Philippines was described as a new species [45]. The groups were selected by age, that is, “younger” females molted into the adult stage about 1 month before experiments started and “older” females ca. 3.5–4.0 months after molt respectively. The age difference between groups was approximately ten weeks. Animals were only considered for experiments with all legs and tarsi completely intact. Prior to the measurements, animals were weighed using a precision scale (Mettler Toledo AG204 DeltaRange, Mettler-Toledo International Inc., USA). Measurements were conducted during daytime, at a temperature of 24.6 ± 1.9 °C and an ambient humidity of 51.0% ± 6.9%. Deceased animals were frozen at −70 °C for subsequent investigation of the tarsal morphology.

### 2 Attachment on a smooth incline

**Figure 2:**
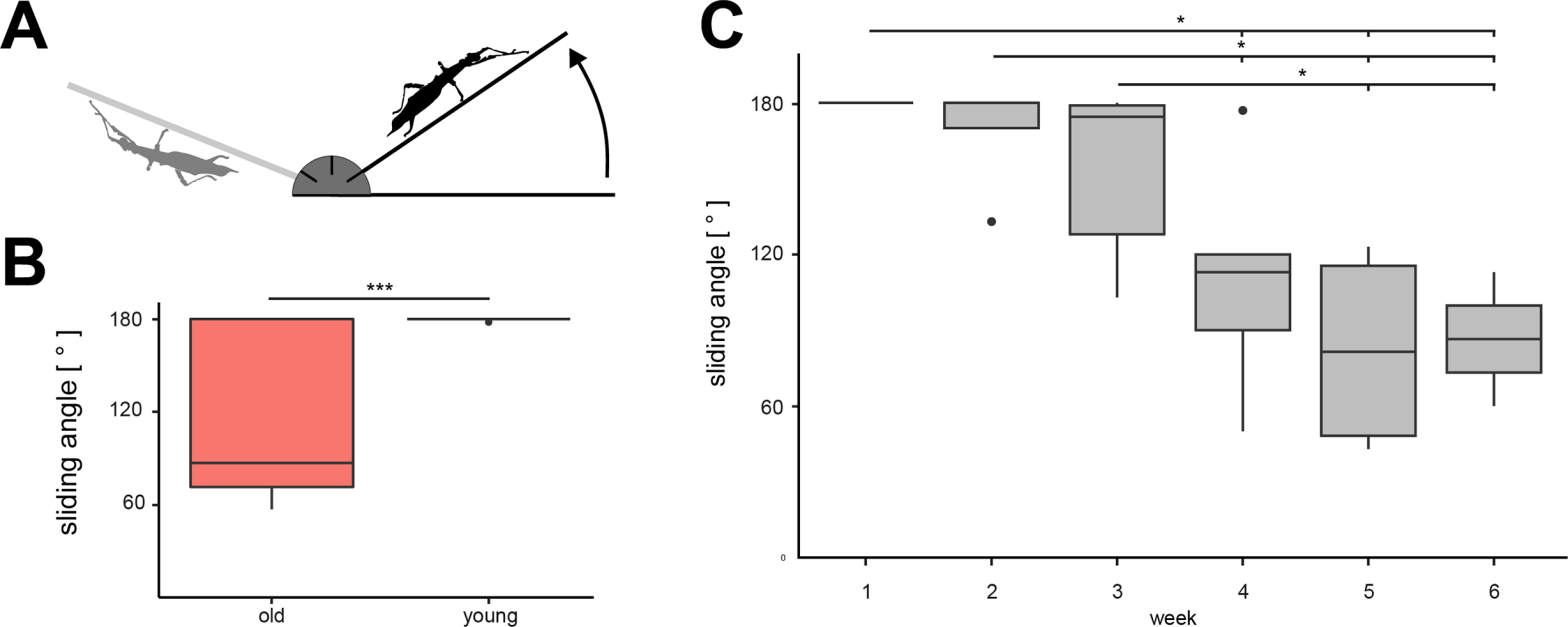
Attachment tests using a tilting platform. (A) Schematic of the experimental setup. (B) Comparison of sliding angles, presented by age (old/young). *** = *p* < 0.001, Wilcoxon rank sum test. (C) Weekly measured sliding angles on glass of the insects while ageing over the course of six weeks. * = *p* < 0.05, One Way Repeated Measures ANOVA/Dunn’s post-hoc test with Holm correction. Boxes cover the interquartile range (IQR) from 25th to 75th percentile, the black line indicates the median. Whiskers extend to 1.5·IQR.

The adhesive abilities on a smooth incline were determined using a custom-made tilting platform following the methodology of Berthé et al. [46] with a glass plate as substrate for attachment. Each animal was placed onto the horizontal glass plate, and the plate was then slowly tilted with an average angular velocity of ca. 3.5° per second until the insect started to slide down or fell off (Figure 2A). The positions and orientations of the animals were standardized, that is, always in the center of the plate with the head facing in the same direction. Values were recorded in intervals of 5°, and the mean of the three measurements was considered for further analysis.

### 3 Force measurements

Attachment force measurements were conducted using a BIOPAC MP100 data acquisition system with a TCI-102 interface (BIOPAC Systems, Inc., USA) and a 100g force transducer (Fort100, World Precision Instruments, Sarasota, FL) using the setup described in Winand and coworkers [37]. We measured pull-off (perpendicular to the substrate) and traction (parallel to the substrate) forces on substrates with three different roughnesses. The substrates were fixed onto a scissor lab jack or a precision slide (Cleveland Lineartechnik GmbH, Löfflingen, Germany). We used glass and epoxy resin [47] replicas of substrates with 1 µm and 12 µm roughness according to Büscher and Gorb [33] to test for differences in the attachment performance on different degrees of surface roughness during ageing. This range of substrate roughness was selected to test for different aspects of the functionality of the attachment pads without major influences of the claws [33,37]. On smooth surfaces (0 µm), smooth pads generate proper contact with the surface. Microrough surfaces interfere with the contact formation of smooth pads; however, the dimension of the nubs on the euplantulae yield different responses to the roughness because fine roughness (1 µm) matches the size of the tips and course roughness (12 µm) matches the size of the entire nubs [33]. The combination of these three levels of roughness was used to investigate potential effects in the three mentioned perspectives of the attachment pads. For details on the fabrication process, the roughness parameters, and the contact angles of the substrates, see [12]. Per trial, the respective force was measured three times per animal and substrate. The order of the substrates was randomized for each direction (pull-off and traction forces) and animal. The insect was anaesthetized with carbon dioxide for 10–15 s before being connected to the force transducer by a string of fishing line (0.18 mm) at the mesothorax at the estimated center of mass [33,48]. The animals were allowed to recover for some minutes until they were responding to being lifted off the substrate with leg movements.

Time–force curves were recorded using AcqKnowledge (3.7.0, BIOPAC Systems, Inc., USA) while moving the substrate and platform manually with steady speed in the required direction. The maximum traction or pull-off force was recorded (see [33]). The means of the three measurements per trial were used for data analysis to reduce intra-individual variance. As one old female deceased within the experimental time, the sample size was 15 for both groups regarding pull-off forces and for the young group regarding traction forces; the sample size was 14 for traction force measurements in the old group. A list of all measurements of attachment forces and body weights is included in Supporting Information File 1.

### 4 Attachment over time

To further investigate the relation between progressing age and pull-off/traction force performance, six of the younger animals were used for further experiments. The abovementioned attachment measurements (see sections 2 and 3) were repeated once a week for six consecutive weeks. The measurements started ca. 1.5 months post adult molt. The order of substrates and the direction to be measured first were randomized per animal and week.

### 5 Light microscopy

The tarsi of all animals were documented postmortem using a stereo microscope (Nikon SMZ745T, Nikon Corporation, Tokyo, Japan). Pictures were taken using a Sony DSC-RX0 (Sony Group Corporation, Tokyo, Japan) equipped with a C-mount adapter using a RX0 Mod Kit (Back-Bone Gear Inc., Ontario, Canada). Frozen animals were allowed to thaw, and tarsi were removed for examination. Stacks of images were taken from different focus planes and combined subsequently. Images were processed using Adobe Photoshop v24.7 and Adobe Lightroom Classic 12.0 (Adobe Inc., San Jose, USA). After focus stacking and cropping, clarity and contrast were adjusted.

### 6 Widefield fluorescence microscopy (WFM)

Autofluorescence signals of insect cuticle can be used to investigate the material composition of the arthropod exoskeleton [49]. To scan for differences in the fluorescence, a selection of tarsi across all age groups was examined using WFM. Freshly molted adult and subadult individuals were acquired from laboratory stock and used for imaging as well. Three individuals were chosen for each age group.

Tarsi were cut off at the tarso-tibial joint and transferred into 1.5 mL solution of phosphate-buffered saline (PBS) and Triton TM-X100 (Sigma-Aldrich, St. Louis, USA) for 30 min to reduce surface tension and enable proper glycerin coating. Afterwards, samples were rinsed three times in glycerin and then fully submerged in glycerin and covered with a high-precision cover slip (Carl Zeiss Microscopy GmbH, Jena, Germany).

Images were taken using a Zeiss Axioplan microscope and an AxioCam MRc camera with the AxioVision software (v. 4.8.2) (Carl Zeiss AG, Oberkochen, Germany). The tarsi were examined at 5× magnification. Sets of excitation and emission filters were used according to [50].

### 7 Confocal laser scanning microscopy (CLSM)

A confocal laser scanning microscope (Zeiss LSM 700, Carl Zeiss Microscopy GmbH, Jena, Germany) with stable solid-state lasers (wavelengths 405, 488, 555, and 639 nm) and the corresponding band- and longpass emission filters (BP420–480, LP490, LP560, and LP640 nm) was used to obtain detailed information about the autofluorescence of the cuticle [50]. The samples were prepared the same way as for WFM imaging (see section 6). One tarsus per respective age group was examined (subadult, freshly molted adult, young, and old). The ZEN2008 software (Carl Zeiss AG, Oberkochen, Germany) was used to generate maximum intensity projections.

### 8 Scanning Electron Microscopy (SEM)

For inspection of the tarsal morphology of different age groups, samples were chosen after CLSM to compare regions of interest, such as altered autofluorescence or damage. Selected tarsi were transferred from glycerin into 50% ethanol via a gradual series of glycerin (descending) and ethanol (ascending) mixtures. Afterwards, samples were dehydrated in an ascending ethanol series and dried using a Leica EM CPD300 (Leica, Wetzlar, Germany) critical point drier. The tarsi were mounted on SEM stubs and sputter-coated with 10 nm gold–palladium in a Leica Bal-TEC SCD500 (Leica Camera AG, Wetzlar, Germany) coater. A Hitachi TM3000 (Hitachi Ltd. Corporation, Tokyo, Japan) scanning electron microscope was used to document the tarsal morphology at 15 kV acceleration voltage.

### 9 Data analysis

Data analysis was performed in the R environment [51] using R Studio [52]. Data was tested for normal distribution and homoscedasticity using Shapiro–Wilk test and Levene’s test, respectively, the latter from the “car” package [53]. Performance by direction and substrate for time series over six weeks was compared with One Way Repeated Measures Analyses of Variance (ANOVA) and Tukey’s post-hoc test or Friedman’s Repeated Measures ANOVA and Dunn’s post-hoc test with Holm correction (“FSA” package, [54]), depending on the results of the preassumption tests. For pull-off and traction forces of old and young animals, Kruskal–Wallis One Way ANOVA and Dunn’s test with Holm correction were used instead. Wilcoxon rank sum test was used for the comparison of sliding angles between old and young adult animals, according to the results of the Shapiro–Wilk test.

## Results

### Attachment on a smooth incline

On the tilting platform, young adult animals started sliding or detached from the substrate at 179.87° ± 0.52° (mean ± SD), whereas older animals lost grip at 118.87° ± 54.98° (Figure 2B). Instances where angles of 180° were reached did not cause the animals to slide. Despite the amount of variation among sliding angles on glass in older animals (range: old = 123.33°, young = 1.67°), the sliding angles of young adult animals were significantly higher than those of old adult animals (Wilcoxon rank sum test, *U* = 40.500, *p* < 0.001).

The attachment ability of the younger adult animals (*N* = 6) that were tested over the range of six consecutive weeks faded gradually (Figure 2C). The maximum angle at which the animals started sliding off the incline declined significantly (One Way Repeated Measures ANOVA, *F* = 12.299, d.f. = 5, *p* < 0.001). In the first three weeks, the mean sliding angle decreased slowly (week 1: 180.0° ± 0.0°; week 2: 168.6° ± 20.4°; week 3: 154.2° ± 36.0°). The mean sliding angles in these three weeks did not differ significantly from each other (Dunn’s test with Holm correction, all *p* > 0.050). The mean sliding angle dropped to 110.0° ± 46.4° in week 4, which was significantly lower compared to the first two weeks (Dunn’s test with Holm correction, all *p* < 0.05), but not different from week 3 (Dunn’s test with Holm correction, *p* = 0.092). The sliding angles further decreased in week 5 (82.3° ± 41.69°) and week 6 (86.5° ± 37.5°), resulting in significantly lower sliding angles compared to the weeks 1–3 (Dunn’s test with Holm correction, all *p* < 0.050). From week 5 to week 6, sliding angles remained similar (Dunn’s test with Holm correction, *p* = 0.752). Variance increased over time.

### Attachment forces

#### Attachment performance of young and old animals

**Figure 3:**
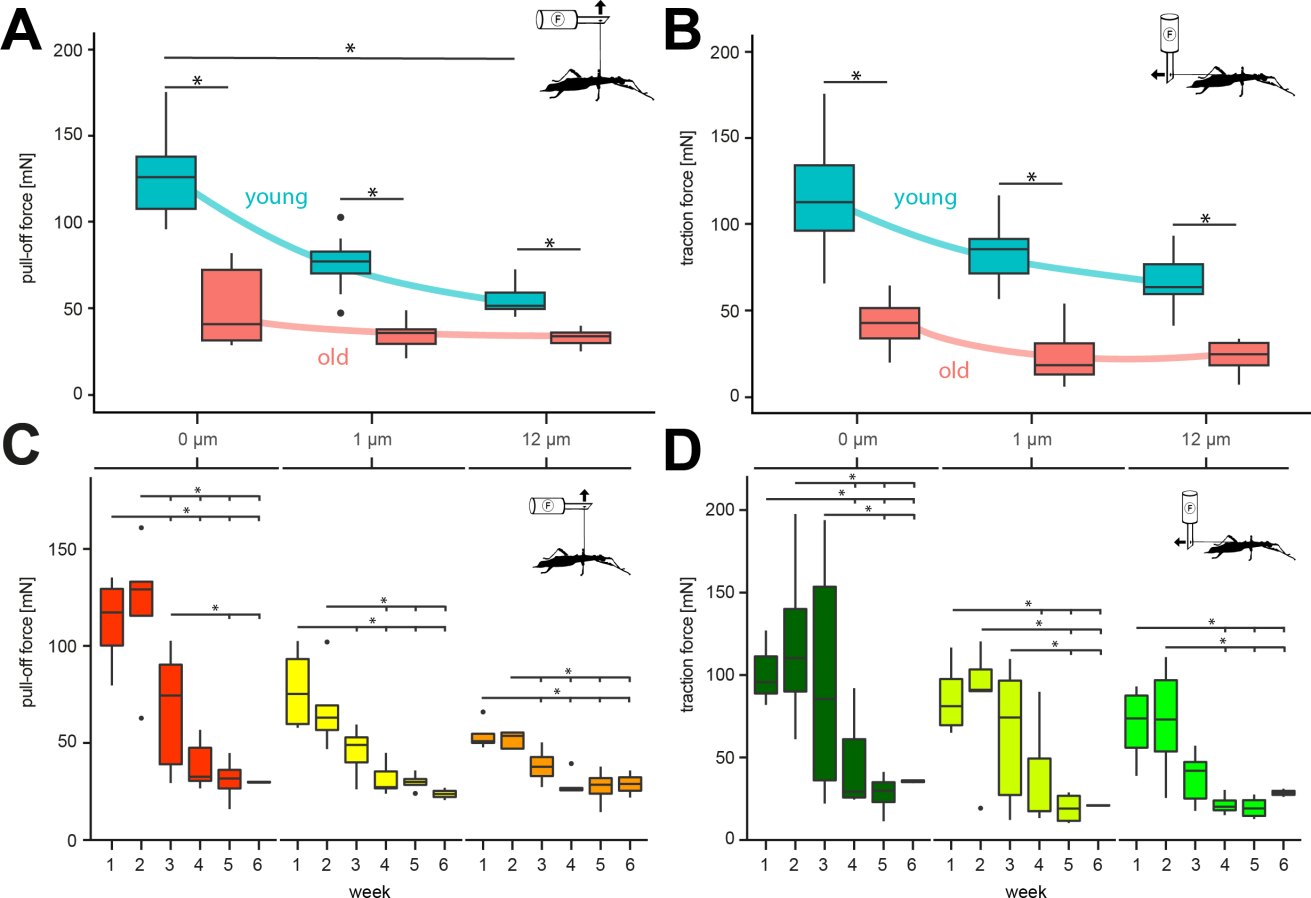
Attachment force measurements. (A, B) Comparisons of attachment forces of old and young adult females on substrates with different roughness. (A) Pull-off forces and (B) traction forces. * = *p* < 0.05, Kruskal–Wallis ANOVA on ranks/Dunn’s post-hoc test with Holm correction. (C, D) Change of attachment forces of adult females on substrates with different roughness over the course of six weeks. Colors in C and D represent the same substrate as indicated on the top of the graph. (C) Pull-off forces. (D) Traction forces. * = *p* < 0.05, Kruskal–Wallis ANOVA on ranks/Dunn’s post-hoc test with Holm correction. Boxes cover the interquartile range (IQR) from 25th to 75th percentile, the black line indicates the median. Whiskers extend to 1.5·IQR.

In the pull-off direction (Figure 3A), both age and substrate had some effect on the measured forces (Kruskal–Wallis ANOVA on ranks, *H* = 66.677, d.f. = 5, *N*_young_ = 15, *N*_old_ = 15, *p* ≤ 0.001). In young adult animals, pull-off forces differed significantly between the three substrates (Dunn’s test with Holm correction, all *p* < 0.010) and were highest on glass (124.38 ± 22.55 mN), less high on 1 µm (75.48 ± 13.51 mN), and lowest on 12 µm rough substrates (54.85 ± 8.72 mN). No significant effect was found between substrates in older animals (Dunn’s test with Holm correction, all *p* > 0.050). The pull-off forces for old animals were also highest on glass (50.37 ± 21.46 mN), less high on 1 µm (34.09 ± 8.48 mN), and lowest on 12 µm rough substrates (32.46 ± 4.59 mN). Younger animals performed significantly better on all substrates compared to older animals on the same substrate (Dunn’s test with Holm correction, all *p* < 0.050).

Traction forces (Figure 3B) showed relationships qualitatively similar in different animals to pull-off forces. In younger adult animals, traction forces were significantly influenced by the substrate roughness (Kruskal–Wallis ANOVA on ranks, *H* = 72.314, d.f. = 5, *N*_young_ = 15, *N*_old_ = 14, *p* ≤ 0.001). Similar to the pull-off forces, the highest values were obtained on glass (122.31 ± 36.48 mN), lower forces on 1 µm (82.38 ± 15.25 mN), and lowest on 12 µm rough substrates (66.78 ± 14.8 mN). The traction forces on the three substrates differed significantly from each other in young adult animals (Dunn’s test with Holm correction, all *p* < 0.050). Traction forces of older animals were influenced by the substrate as well (Kruskal–Wallis ANOVA on ranks, *H* = 72.314, d.f. = 5, *N*_young_ = 15, *N*_old_ = 14, *p* ≤ 0.001). The forces were significantly higher on glass (43.13 ± 14.2 mN) compared to 1 µm (21.48 ± 14.26 mN) and 12 µm rough substrates (22.93 ± 8.74 mN) (Dunn’s test with Holm correction, all *p* < 0.050). No significant difference was found between 1 and 12 µm rough substrates (Dunn’s test with Holm correction, all *p* > 0.050). Differences between age groups on the same substrate were all significant (Dunn’s test with Holm correction, all *p* < 0.050).

Signs of ageing were apparent during the attachment force measurements. Older animals were observed to establish less rigorous contact of their tarsi with the substrates at some occasions. During traction force measurements, sometimes tarsi were not aligned with the direction of the pulling movement and were sliding more easily compared to other tarsi. However, these problems with contact formation were not persistent throughout the experiments and occurred only from time to time.

#### Attachment forces over time

Variances of pull-off forces were higher on glass and 1 µm roughness during the first weeks and decreased towards the fifth and sixth week, whereas results on 12 µm roughness showed the least variance across the time span. All three substrates revealed significant differences over time (RM ANOVAs, all *p* ≤ 0.001). The pull-off force on glass (RM ANOVA, *F* = 22.437, d.f. = 5, *p* ≤ 0.001) gradually decreased from 112.34 ± 24.83 mN in week 1 to 29.790 ± 0.56 mN in week 6. The changes of pull-off force on glass between week 1 and weeks 3–6 (Tukey’s tests, all *p* < 0.005), between week 2 and weeks 4–6 (Tukey’s tests, all *p* < 0.001), and between week 3 and weeks 5–6 (Tukey’s tests, all *p* < 0.030) were found to be significant. On 1 µm roughness (RM ANOVA, *F* = 14.346, d.f. = 5, *p* ≤ 0.001), the forces were lower than on glass in week 1 (77.72 ± 22.11 mN) and declined to 23.72 ± 4.49 mN in week 6. The changes of pull-off force on 1 µm between week 1 and weeks 3–6 (Tukey’s tests, all *p* < 0.003) as well as between week 2 and weeks 4–6 (Tukey’s tests, all *p* < 0.003) differed significantly. On 12 µm (RM ANOVA, *F* = 15.618, d.f. = 5, *p* ≤ 0.001), the pull-off forces in week 1 were lowest compared to the other substrates (53.88 ± 8.21 mN) but still decreased towards week 6 (28.86 ± 9.83 mN). The changes from week 1 to weeks 4–6 (Tukey’s tests, all *p* < 0.018) as well as from week 2 to weeks 4–6 (Tukey’s tests, all *p* < 0.039) were significant as well. The pull-off forces of the remaining combinations did not differ significantly from each other (Tukey’s tests, all *p* > 0.15).

The mean traction forces declined on all surfaces over time following the same trends as the pull-of forces (Figure 3D). The traction changed significantly over time as well (RM ANOVAs, *F*_glass_ = 16.484, *F*_1 µm_ = 12.540, *F*_12 µm_ = 8.784, all d.f. = 5, all *p* ≤ 0.001). Forces declined from 126.90 ± 54.18 mN (glass), 85.99 ± 23.27 mN (1 µm), and 69.84 ± 24.59 mN (12 µm) to 35.60 ± 1.52 mN (glass), 20.95 ± 0.42 mN (1 µm), and 28.58 ± 3.47 mN (12 µm). For glass, the changes between week 1 and weeks 4–6 (Tukey’s tests, all *p* < 0.003), between week 2 and weeks 4–6 (Tukey’s tests, all *p* < 0.007), and between week 3 and weeks 5–6 (Tukey’s tests, all *p* < 0.003) were significant. On 1 µm roughness, forces changed significantly from week 1 to weeks 4–6 (Tukey’s tests, all *p* < 0.011), from week 2 to weeks 5–6 (Tukey’s tests, all *p* < 0.002), and from week 3 to weeks 5–6 (Tukey’s tests, all *p* < 0.008). On the 12 µm substrate, only changes from week 1 to weeks 4–6 (Tukey’s tests, all *p* < 0.360) and from week 2 to weeks 4–6 (Tukey’s tests, all *p* < 0.044) were significant. The traction forces of the remaining combinations did not differ significantly from each other (Tukey’s tests, all *p* > 0.06).

### Morphological changes

#### Macroscopic changes of attachment devices

**Figure 4:**
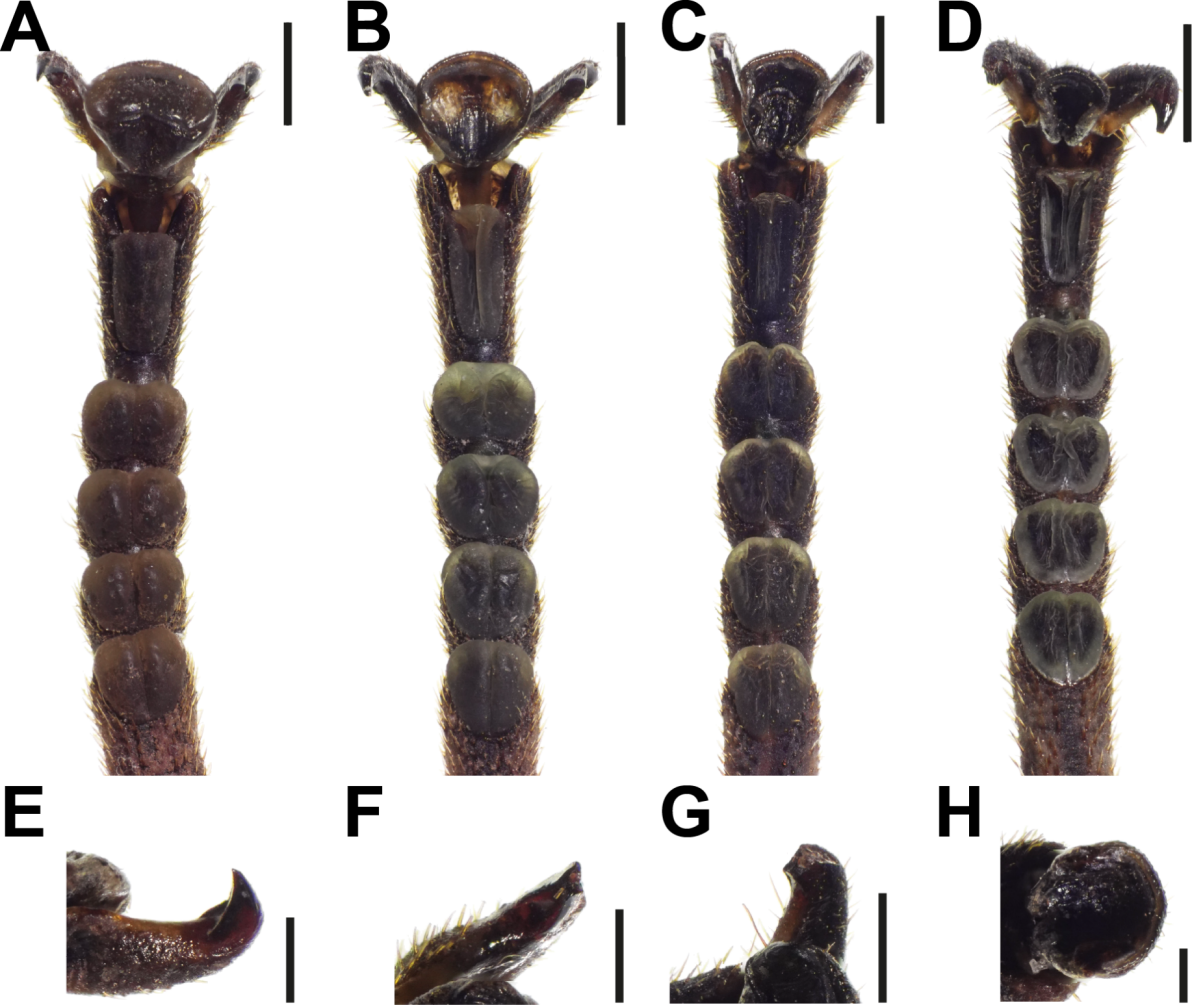
Morphological changes of tarsi during ageing. (A–D) Full tarsi, ventral views. (A) Young adult female with fully inflated arolium and euplantulae. (B–D) Old adult females; different degrees of deflation of arolia and eupantulae are visible. (E–H) Different degrees of claw wear in old adult females, ventral views. (E) No damage visible. (F) Claw tip broken off. (G) Larger fraction of claw broken off. (H) Claws missing. Scale bars: (A–D) 1 mm, (E–H) 300 µm.

All tarsi of *S. aeta* possess five euplantulae on their five tarsomeres and one arolium situated between two claws on the pretarsus (Figure 1). Ageing was mainly visible from the shape of the attachment pads themselves (Figure 4). Observations via stereomicroscopy showed that in younger animals all attachment pads are fully inflated and appear tightly filled with the fluid (Figure 4A). The condition of the attachment pads varied in older animals. Euplantulae and arolia were frequently observed to be sunken in or shriveled and discolored (Figure 4B–D). Additionally, the same pads showed variance in deflation across different specimens or legs of the same animal. Also, the attachment pads differed in the degree of deflation, depending on the tarsal segment they are located on. The degree of deflation of the pads was always higher in the distal ones. The distalmost arolium was most strongly affected by deflation in most of the cases (Figure 4B–D), whereas the degree of deflation in euplantulae differed depending on how distal the particular euplantula was situated on the tarsus (Figure 4C,D). Overall, the extent of deflation varied across the specimens and tarsi of the same animal. However, the deflation was generally strongest for older animals.

Claws were uniform in color, but claw wear was observed to vary across specimens (Figure 4E–H). In general, claw conditions ranged from fully intact (Figure 4E) to completely missing (Figure 4H). Most frequently the claw tips were broken (Figure 4F,G). The wear was strongest in older animals, but observed through all age groups.

#### Material changes

**Figure 5:**
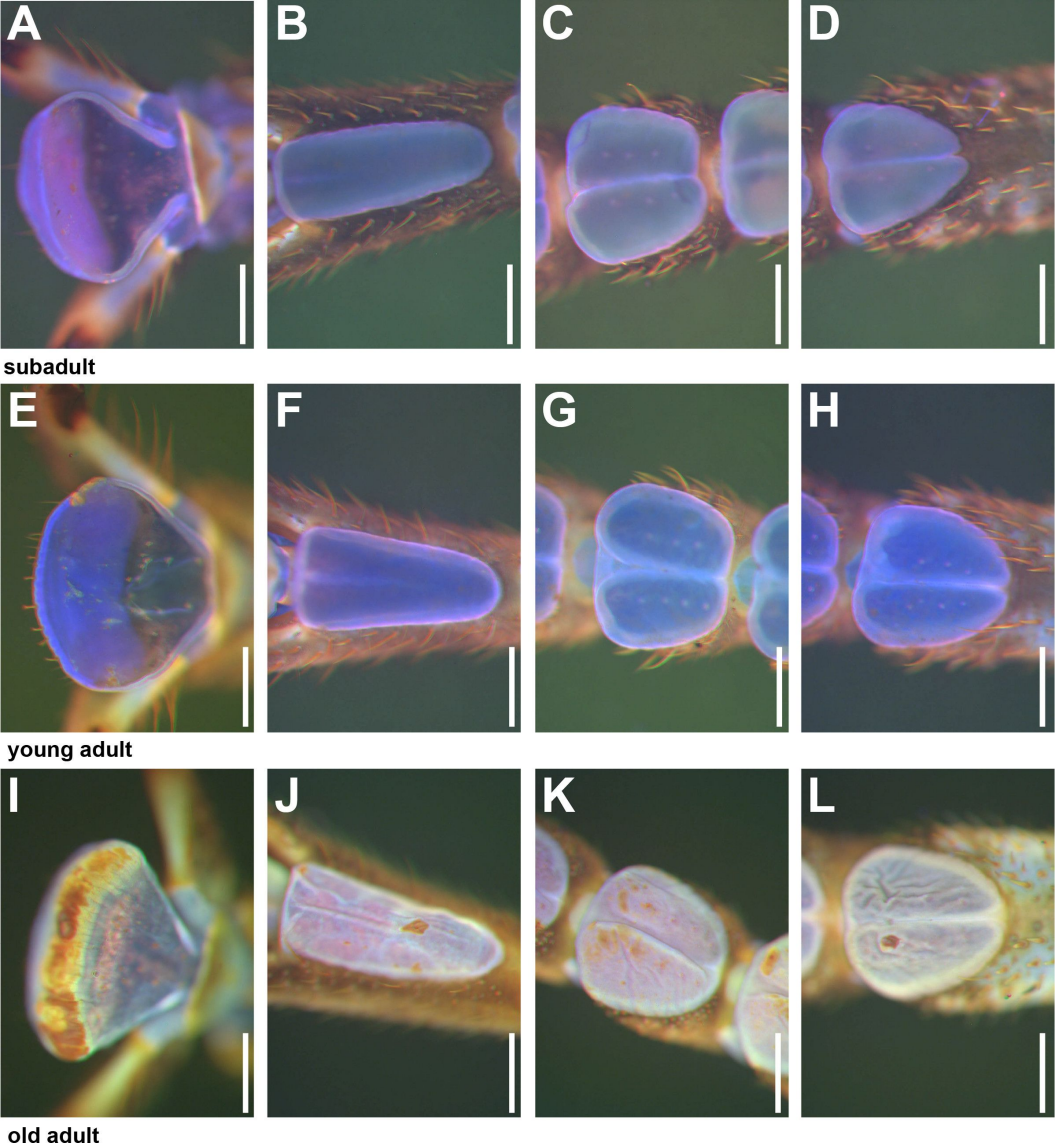
Ventral views of attachment pads obtained from WFM. (A–D) Subadult female. (E–H) Young adult female. (I–L) Old adult female. Images within one row are from different areas of the same sample. Vibrant blue color indicates soft cuticle, dark yellow-red color indicates stiffened cuticle. (A, E, I) Arolia. (B, F, J) Fifth euplantula. (C, G, K) Third euplantula. (D, H, L) First euplantula. Attachment pads show increasing stiffened areas with age and relatively less strong autofluorescence signals of the soft cuticle. Scale bars: 200 µm.

**Figure 6:**
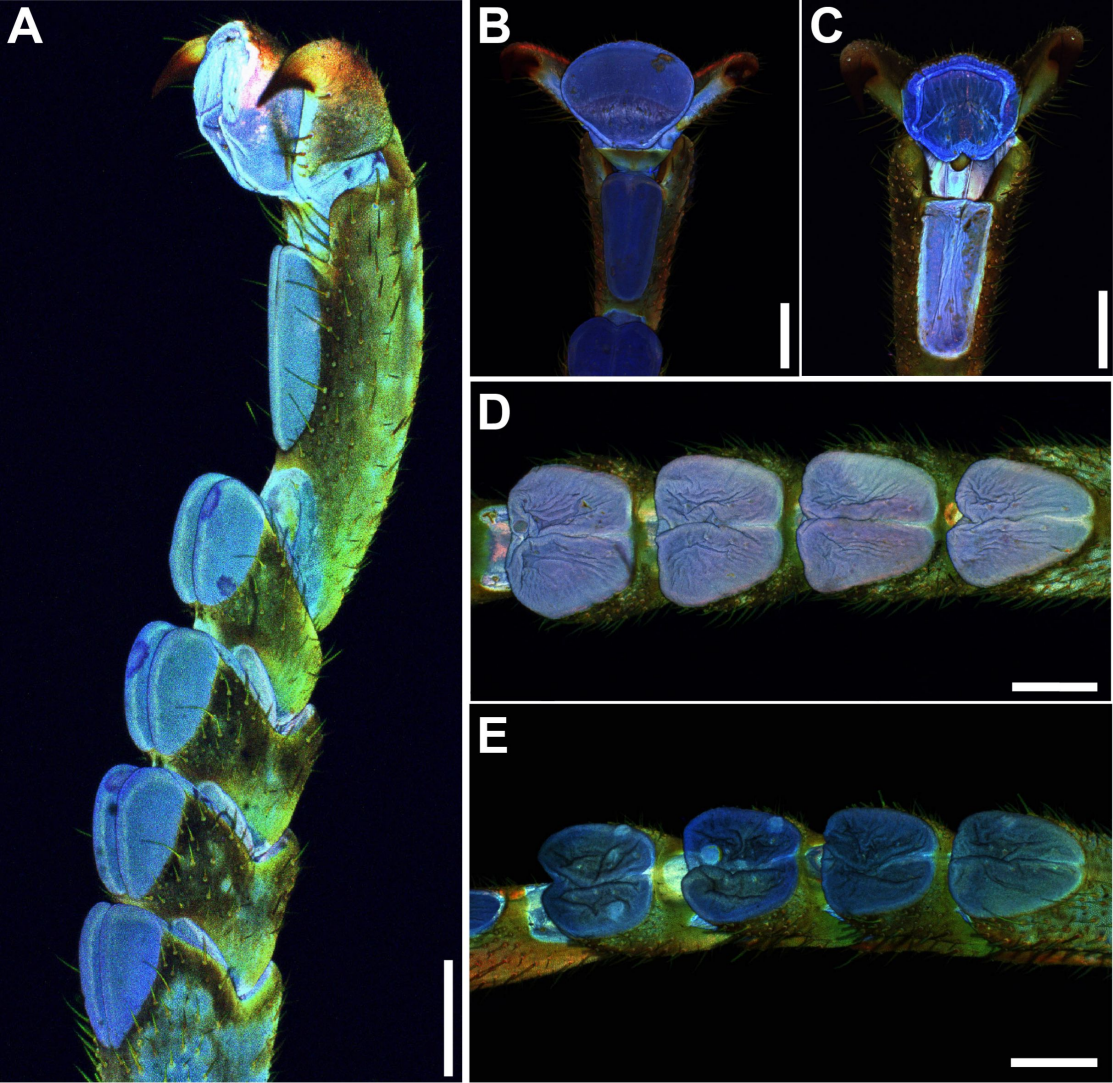
Maximum intensity projections of tarsi in different age groups obtained by CLSM. (A) Tarsus of subadult female, lateral view. (B) Young adult pretarsus, ventral view. (C) Old adult pretarsus, ventral view. (D) Young adult euplantulae, ventral view. (E) Old adult euplantulae, ventral view. Colors indicate relative material autofluorescence. Blue signals indicate resilin-containing weakly sclerotized cuticle, and green and red signals indicate stronger sclerotized cuticle. Scale bars: 1mm.

**Figure 7:**
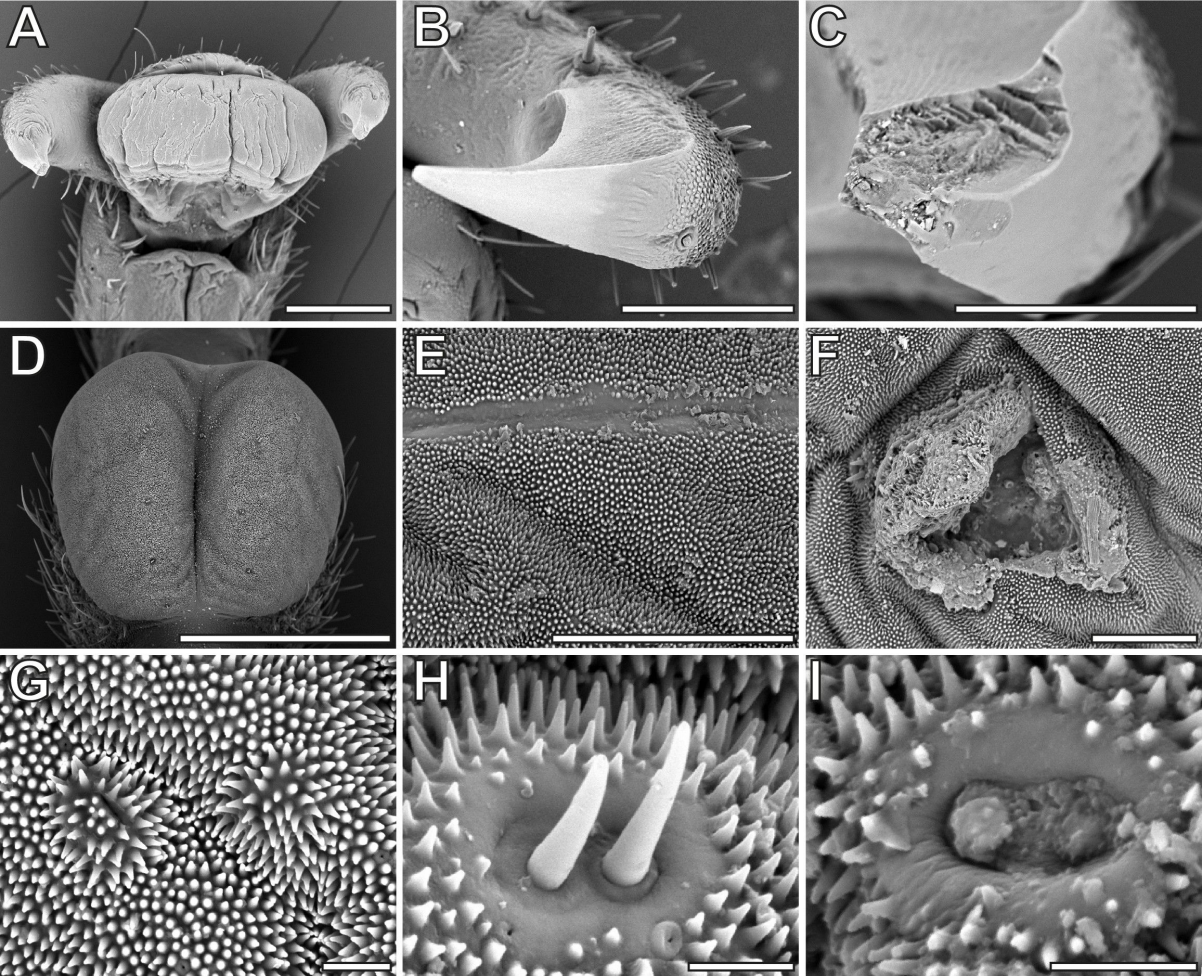
SEM micrographs of ageing effects on the tarsi. (A) Pretarsus of old female, ventral view. (B) Intact claw, young adult female. (C) Broken claw, old female. (D) Intact inflated euplantula, young adult female. (E) Scratches on euplantula, old female. (F) Larger scar on euplantula, old female. (G) Inhomogeneously deformed cuticle of the euplantula, old female. (H) Intact mechanoreceptors, young adult female. (I) Worn mechanoreceptors with detached setae, old female. Scale bars: (A, D) 500 mm, (B, C, E, F) 100 µm, (G–I) 10 µm.

Changes of the cuticle of the attachment pads were investigated via WFM and CLSM. Both methods were used to visualize the autofluorescence of the pad cuticle, which is informative about the cuticle composition, for example, the degree of sclerotization [50,55]. Both methods indicate the degree of sclerotization through the autofluorescence of the materials excited with light of different wavelengths. The detected autofluorescence signals are visualized in different colors according to the excitation wavelength [50]. Blue indicates less sclerotized cuticle, green indicates rather sclerotized cuticle, and red colors indicate strongly sclerotized cuticle [50,55]. The general appearance of the autofluorescence and its distribution was uniform for all tarsi examined and corresponds to the signals known for stick insect tarsi [56]. Differences in color between the pads and the cuticle of the tarsus were clearly visible. The adhesive pads were generally fluorescing blue in both measurements (Figure 5 and Figure 6). Using WFM, the cuticle of the tarsomeres appeared in a yellow-orange color (Figure 5) and showed red and green signals in CLSM (Figure 6), indicating their stronger degree of sclerotization. A double row of dots with red autofluorescence located on the pads along the central groove was visible using WMF (e.g., Figure 5C); it can be assigned to the position of mechanoreceptors (see Figure 7D,H below and also [37]). No differences in the autofluorescence pattern were seen among front, middle, and hind legs.

The blue color of the cuticle of attachment pads appeared more vibrant using WFM in the subadult individuals (Figure 6A–D) and young adult animals (Figure 6E–H) than in the older animals (Figure 6I–K). Because of individual settings for each scan of the CLSM, the colors of the maximum intensity projects are not directly comparable among the images. However, the relative distribution of signals can be informative for the comparison of signs of ageing in combination with shape changes of the attachment pads. The deflation of the euplantulae and arolia of older animals is also visible in WFM (Figure 5I,L) and CLSM (Figure 6C–E). The deflation leads to strongly wrinkled pad surfaces. The tarsi of young adult animals sometimes revealed smaller patches with derived autofluorescence signals on the attachment pads (Figure 5E). Instead of the vibrant blue signal of the surrounding cuticle, some areas appear orange to brown in WMF (Figure 5F) images, or green to red in CLSM (Figure 6B) images, typical for stronger sclerotized cuticle. The size and proportion of such patches was higher on the pads of older animals (Figure 5 I), and large parts of the euplantular area frequently showed an overall reddish hue throughout the pad surface (Figure 5I–L).

#### Microscopic ageing signs

Several further microscopic signs of ageing were visible using SEM (Figure 7). Wrinkles due to deflation of the pads often caused furrows on the surface of arolia (Figure 7A). While the original condition (Figure 7D) of the euplantula exhibits a bilobed inflated pad without major markings, except for the central grove and the nubby attachment microstructure, different wear marks were observed on the euplantulae of older animals. The wear patterns included scarred scratches (Figure 7E), scarred tissue from larger wounds (Figure 7F), and deformations of the pad surface that potentially arose from inhomogeneous changes of the material properties of the cuticle (Figure 7G). Other wear marks were found on the claws (Figure 7C) and on the mechanoreceptors of euplantulae (Figure 7I). While the contact sensilla on the euplantulae are usually found in pairs within groves without micro-ornamentation and are well recognizable (Figure 7H), the setae of the mechanoreceptors were often worn off in older animals (Figure 7I).

**Figure 8:**
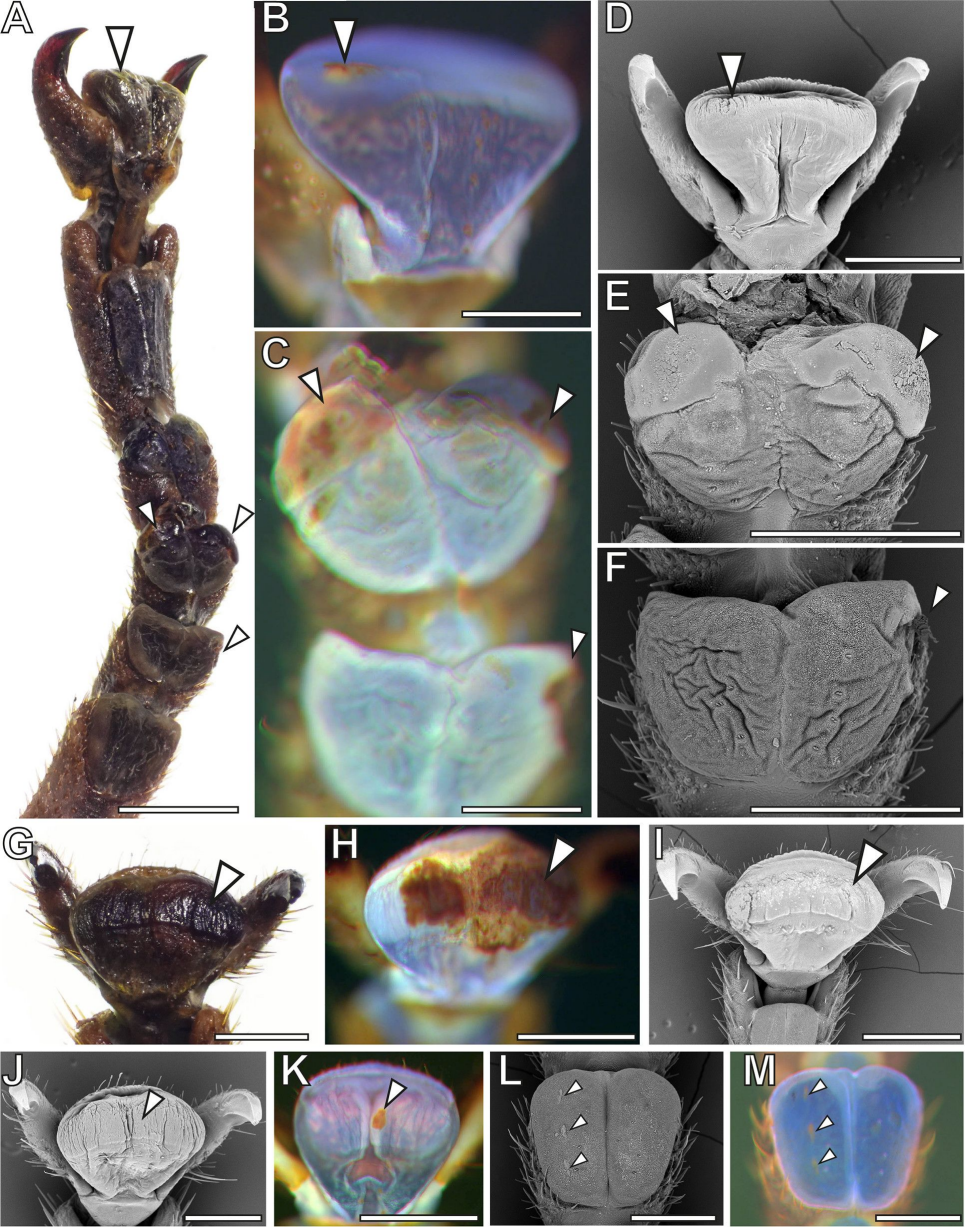
Combination of visualizations of the same attachment organs with different microscopy techniques. A–F, G–I, J–K, and L–M correspond to the same respective tarsi of old adult females. Arrowheads mark areas of concern. (A, G) Stereomicroscopy images showing the appearance of the attachment pads. (B, C, H, K, M) WMF images showing native bluish regions and those stiffened due to the ageing. (D–F, I, J, K) SEM images showing the topography of the surface. Scale bars: (A) 1 mm, (B–M) 500 µm.

Certain changes of the attachment pad cuticle that were not visible using some methods were verified with other microscopy techniques (Figure 8). Larger deformations of the attachment pads, visible by stereomicroscopy (Figure 8A), often appeared dark and brownish in WFM (Figure 8C), which could also be due to contamination. SEM revealed most of such cases as not being caused by contaminations. They rather arose from a strong alteration of the cuticle (Figure 8E), also including changes of the surface topography of the terminal layer of the attachment pad cuticle. Profound hardening of the cuticle could yield an appearance similar to a pad coverage by other substances resulting in dark patches in WFM (Figure 8B,H,K). Such patches usually showed no covering films visible in stereomicroscopy (Figure 8A,G) and SEM (Figure 8D,I,J). Scars on the attachment pads (Figure 8L) also appeared red to brown in WFM (Figure 8M).

## Discussion

### Decay of attachment performance

Older animals showed a decline in adhesive performance similar to cockroaches as previously shown by Ridgel and coworkers [16]. The decline of attachment performance was also measurable here in repetitive tests over a longer time span. Animals lost adhesive abilities on all substrates over the course of six weeks, and their attachment performance converged to the level of the older animals in the first series of experiments. Variation of the attachment performance increased with age, which could be seen in the direct comparison of sliding angles and in the measurements over time. Ageing is a gradual process; hence, the decline of attachment abilities can be expected to be gradual as well and to show intraspecific variation [17]. A difference in activity of the animals was also noticeable during the experiments. The young individuals were more active, whereas the old animals took longer to recover from anesthesia (not quantified). Ridgel and Ritzmann [5] also detected a decrease of around 50% in walking speed of aged cockroaches. This matches the proposed loss of muscle fibers with age, leading to muscle atrophy [16].

### Roughness dependence of attachment performance decay

The performance of insect attachment pads recorded on a smooth surface is usually higher than on microrough surfaces [14], and asperity sizes around 0.3–1.0 µm have been confirmed to be a critical roughness at which adhesion is the lowest [33,57–60]. On substrates with higher roughness, claws are used for mechanical interlocking. However, this effect requires the substrate asperities to be at least the size of the claw tip diameter [61]. Claw involvement was excluded herein as surface roughnesses were chosen accordingly. Winand et al. [37] showed that a roughness of 12 µm is at the lower end of the effective claw usage for *S. aeta*. This allowed us to compare the age-related performance differences of the attachment pads.

Büscher und Gorb [33] compared the adhesive performance of two species of stick insects including *S. aeta*. The results therein for the same species were similar to the measurements of young adult animals herein and revealed generally higher pull-off and traction forces on a smooth surface, whereas the forces on microrough surfaces were lower [33]. The protuberances on the euplantulae of the stick insect *Carausius morosus* Brunner von Wattenwyl, 1907 (Lonchodidae) have a tip diameter of about 0.5 µm [31] and a ratio of length to width comparable to the nubs of *S. aeta* [37]. Apparently, the ratio of the nubs, compared to the surface asperities, makes better contact on a 1 µm rough surface, if compared to a 12 µm rough surface in younger adult individuals. This effect vanished for older adult animals, as attachment forces became more similar on all three substrates. No change of nub morphology was observed in older animals (Figure 7G,I), but a change of stiffness of the nubs could potentially affect their functionality as well. Nevertheless, pad compliance and deflation likely have a stronger effect in this experiment as discussed below. Other studies reported lowest forces on 1 µm and higher forces on 12 µm roughness for adhesion and traction in other arthropods [59–60]. This effect was presumably due to differences in micromorphology of their adhesive systems. Despite the gradual decay of attachment ability, no safety factors (attachment force per weight force) below 1 were observed, even in older animals; according to Pillai et al. [62], this would have been an indicator for the failure of the adhesive system to statically hold the insect’s weight on the ceiling. Apart from insects, roughness plays a role in adhesion of non-arthropods as well. Roughnesses of 100–300 nm had the largest attachment-reducing effect for both single setae and whole geckos in experiments with the species *Gekko gecko* (Linnaeus, 1758) [63]. Similar to insects, attachment performance in geckos can vary between species on different roughnesses, depending on the morphology of their adhesive systems [64–67]. Similar to the results shown here, geckos face similar challenges to sustain attachment during ageing [64], that is, damage, contaminations, and changes of material properties of the integument of the attachment pads. Geckos, however, continuously shed their skin throughout their life, in contrast to insects; this was shown to enable regeneration of the adhesive properties of the attachment system to some extent [62].

### Claw wear

Claws did not play a role in our attachment experiments, but they were morphologically investigated as part of the tarsus as well. No link between age and claw wear could be established as claw wear usually happens abruptly at single instances and accumulates over time [68]. Damage on the claws consequently rather indicates whether a particular individual claw experienced sufficient stress to be damaged than informs about the age, besides the fact that the longer life time potentially leads to the higher probability of such events. Claws are therefore unsuitable to determine the age of *S. aeta*. Some arthropod claws, such as those in ticks [69], have been shown to contain small amounts of resilin, an elastomeric protein providing flexibility in cuticle composites [70]. Voigt and Gorb [69] also suspect resilin to occur in other arthropod claws as well, but melanization impedes investigation using fluorescence microscopy. Resilin-containing structures within the claws could potentially work as damping mechanisms to reduce wear and risk of damage [69]. Claws are presumed to be more resistant to wear than the soft and pliable adhesive pads [68,71]. Most claw breakages were observed at the tips; the tips have to withstand the greatest stresses, which mostly occur in single events, rather than in normal wear [68]. Further studies could explore the role of fatigue of claw material and its effect on the mechanical properties.

### Pad compliance

There are several possible ways in which attachment abilities could be affected by ageing. Compliance of the attachment pad to the substrate plays a significant role for the performance. The compliance of the attachment pad surface can be negatively affected by changes of the material properties of the cuticle and through structural damage of the surface, leading to obstacles for contact formation at the interface between the pad and substrate [11]. Ridgel et al. [16] noticed dry and dark pads in aged cockroaches, but they could not explain why the pads changed appearance and properties. Zhou et al. [17] assumed sclerotized scars to negatively impact pad compliance as such injuries accumulate with age. This effect was also found in different species of tree geckos [62]. A decrease in clinging ability in geckos was recorded with time passed since the last shedding. In contrast to insects, the geckos were able to recover their adhesive ability by molting, which repaired the damage inflicted by day-to-day use and substrate contact. We observed areas on the attachment pads of old adult *S. aeta,* using WFM and CLSM, that showed changes of the pad cuticle (Figure 5 and Figure 8). Some of these areas, appearing darkened using WFM (e.g., Figure 5K) or reddish using CLSM (Figure 6B), are indicative for changes of the material properties of the cuticle. The autofluorescence correlates with the degree of sclerotization [50,70], and stronger cross-linking usually results in stiffer cuticle. As stiffer cuticle is less compliant, and the lower resulting actual contact area leads to lower attachment performance [72–74]. Most flexible cuticle consists at least partially of resilin [50,75–76], which needs water as a plasticizer to retain its extraordinary mechanical properties [70]. As the water evaporates, resilin becomes brittle and less resilient. Pad cuticle was also found to be more prone to evaporation than the leg cuticle [9], which could amplify the resilin degeneration due to sclerotization. Many of the regions of derived autofluorescence on the arolia and euplantulae did not show structural changes in SEM (Figure 8G–K) and likely represent areas of stiffened pad cuticle. Other ageing marks with derived autofluorescence revealed signs of persistent damage to the pad cuticle (Figure 7 and Figure 8). Such dark spots, observed using both fluorescence techniques, are likely scars resulting from repaired damage of the pad cuticle [17]. Abrasion of attachment pad cuticle can be repaired in insects. If the damage is superficial, epicuticle can be restored by depletion of waxes [18,77], but stronger damage results in sclerotization of the wound due to phenolase activity involved in the wound closure [78]. Such sclerotized scars do not only reduce the stiffness of the repaired area; they also cause structural obstacles that interfere with contact formation and reduce attachment performance [17].

### Pad deflation

Besides material properties of the cuticle and microscopic surface features, further effects on attachment performance are likely results of the geometry of the attachment pads [43]. In their study, Ridgel and Ritzmann [5] proposed two ways in which age might affect attachment. They assumed either vascular insufficiency or degeneration of the tracheal system to be responsible for cockroach tarsus degeneration. Stiffer and dryer pads might be results of such ageing processes. Tracheal degeneration could lead to leg tissue dying because of a lack of oxygen and, therefore, might also influence the adhesive pads and their performance. The suspected vascular insufficiency ties into the hemolymph pressure. In the insect circulatory system, the low pressure is kept up by the heart and muscular activity [5,79]. The legs function as a terminal end in the circulatory system and often have accessory hearts to enable hemolymph flow [79]. If age has an effect on a stick insect’s cardiac system, any impairment could additionally decrease the initially low hemolymph pressure. The observed stronger effect of ageing on the more distal regions of the tarsus in *S. aeta* could support the hypothesized influence of lack of the hemolymph support; more distal regions are likely stronger affected by this effect because of their peripheral connection to the circulatory system [79]. A decrease in spontaneous activity has also been reported from senescent insects [16,80]. Less activity would also mean less circulatory support by muscular activity, intensifying the circulatory problems. A relatively large fraction of the tarsus is filled with hemolymph [30], including the volume of the attachment pads [56,81].

In contrast to other insects, such as hymenopterans [82–84], the expansion of the arolium in stick insects is not supported by internal sclerites; instead, it results from the internal pressure within the pad similar to other polyneopteran insects [30,85].

Dening et al. [43] showed that the inflation degree of attachment devices in different animals and artificial attachment devices can play a role in the adhesion control. High pressure within the pad reduced the contact area with the substrate because of the curvature of the pad, and reduced inflation led to larger contact area and increased adhesive performance [43]. As the inflation is achieved by an increased hemolymph pressure in the pads of *S. aeta*, a decline in hemolymph supply to the pads would reduce inflation. The strong extent of deflation visible in these pads might lead to a decrease in adhesive properties due to the formation of folds in the pad surface, resulting in reduced actual contact area, in contrast to the findings of Dening et al. [43]. Tracheal degeneration could further harm the tarsal organs as a lack of oxygen could lead to damage in tissues. Loss of tissues, for example, exocrine cells within the arolium [81], could potentially also influence fluid production within the arolium. These exocrine cells produce adhesive secretions that play different roles in adhesive systems [11,40,85]. In stick insects, such fluids consist of a watery and a lipid phase [86] and, besides interfacial effects, contribute to the shape and curvature of the terminal layer of the attachment pad [43]. Another factor influencing effective stiffness of the cuticle is caused by depletion of these adhesive fluids. Several steps in quick succession were found to dry out the pad cuticle, making it less flexible and providing reduced attachment [40]. Jiao et al. [73] also reported desiccation and depletion of pad fluid to reduce adhesion in excised tarsi.

Pad deflation could also have negative influence on sensory feedback. The mechanoreceptors on the pads of stick insects, which provide feedback about substrate contact [37], usually occur solely on attachment pads with nubby microstructures and only rarely on smooth eupantulae [34]. The setae of mechanoreceptors are usually mounted in a flexible membrane, which also contains resilin [87]. The combination of a changed pad shape and less flexible membranes surrounding the mechanoreceptors’ setae might impair the function and, therefore, reduce the information the animal is able to receive. In their paper concerning ageing cockroaches, Ridgel et al. [16] propose this lack of sensory information to negatively impact the ability of old cockroaches to walk up an incline. It seems plausible to assume that the walking speed might also be affected by poor sensory feedback.

## Conclusion

An effect of age on the attachment abilities of stick insects was found. Attachment and friction forces declined with age on both rough and smooth surfaces. Microscopy investigations revealed deflation of the attachment pads and signs of cuticle hardening, both decreasing pad flexibility and the ability of contact formation to the substrate. The changes observed in the pads of old individuals probably arise from desiccation of the pads and the cuticle, possibly caused by an impaired circulatory system and oxygen deficiency in the tarsus. The effects, such as material desiccation (pads, resilin patches, and membranes), presence of scars on the pad surface, oxygen and hemolymph deprivation, likely reinforce each other. Further experiments could explore more ageing-related effects to gain insights into the processes of attachment ability decay in insects and, thus, potentially improve sustainability of artificial biologically inspired engineering gripping systems. Such studies could include the role of hemolymph pressure for attachment control and the influence of hemolymph within the attachment pads on cuticle hydration and on the production of adhesive fluid.

## Supporting Information

Supporting Information includes the raw data for all experiments, that is, pull-off forces, traction forces, and attachment angles for all animals, as well as their respective weights.

File 1 Raw experimental data.

## Data Availability

All data that supports the findings of this study is available in the published article and/or the supporting information to this article.
